# The case of a 40‐year‐old man with headaches, memory changes, and acute ischemic stroke

**DOI:** 10.1002/acn3.51422

**Published:** 2021-07-24

**Authors:** Gordon Smilnak, Siyuan C. Liu, Christina M. Lineback

**Affiliations:** ^1^ Department of Neurology Northwestern University Feinberg School of Medicine Chicago IL USA

## Summary of case

A 40‐year‐old right‐handed male with a past medical history of HIV well‐controlled on antiretrovirals (last CD4 count 834, absent viral load) presented with headaches, memory changes, and 2 days of right‐sided numbness and weakness.

**Figure 1:**
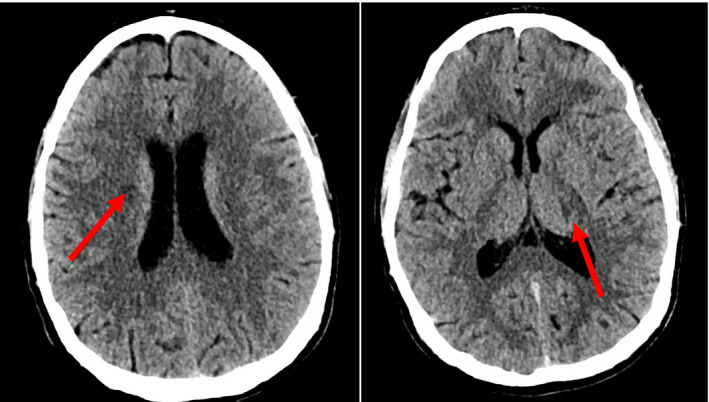
CT Brain without contrast. Obtained during initial workup for the patient showing a poorly defined area of hypoattenuation in the right corona radiata that extends to the lateral margin of the body of the right caudate nucleus. There is a suggestion of a small linear area of hypoattenuation in the lateral aspect of the left side of the thalamus.

**Figure 2:**
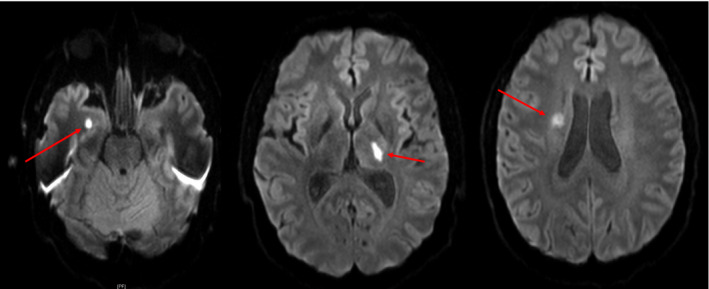
MRI brain without contrast, diffusion‐weighted sequence (DWI). There is restricted diffusion in the left thalamus and in the bilateral anterior temporal lobes that could represent acute infarcts. Late subacute to chronic infarct in the right corona radiata and the superior right basal ganglia.

**Figure 3:**
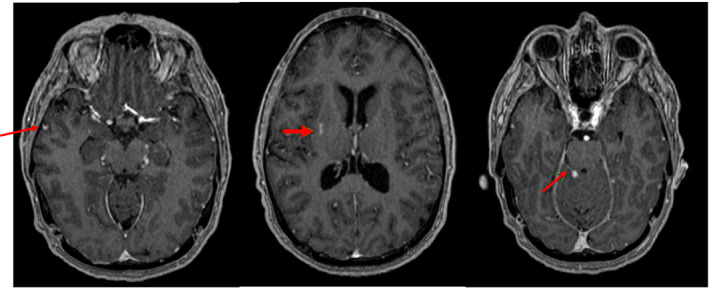
MRI brain with contrast, post‐contrast T1 weight sequences. There are multifocal T2/FLAIR hyperintensities in the right superior cerebellum, left thalamus, bilateral temporal lobes, and right basal ganglia, all associated with mild parenchymal or leptomeningeal enhancement.

He had gradual onset of intermittent headaches over the last month. The headaches were throbbing and, at times, holocephalic. The severity of the headaches worsened over the course of a month. There were no clear triggers to these headaches or preceding aura. There was no associated nausea or vomiting or positional component. The patient has no prior history of headaches. Two days prior to admission, the patient awoke with right‐sided numbness and weakness that started in his arm and leg. He noted difficulty writing with his right hand. His friend noted that the patient had been slurring his words in the last 2 days and also that the patient appeared confused over the same time course (Figs. 1, 2, 3).

An MRI on admission demonstrated multifocal acute infarcts and areas of enhancement. A lumbar puncture demonstrated a positive CSF VDRL, concerning for meningovascular syphilis. The patient was treated with 14 days of penicillin and his symptoms of headache and fatigue improved significantly. He underwent physical therapy for his right‐sided weakness. After the initiation of treatment, he denied any new neurologic symptoms. Meningovascular syphilis is more common in HIV and often is preceded by a prolonged prodrome of headache, confusion, and meningismus, such as in our patient.

## Diagnosis

Meningovascular syphilis

## Take‐home points

1. In patients who are immunocompromised or with limited cardiovascular risk factors, infectious etiology should be considered on the differential of acute ischemic stroke.
2. Meningovascular syphilis is more common in HIV and often is preceded by a prolonged prodrome of headache, confusion, and meningismus.
3. Successful diagnosis of meningovascular syphilis requires a high degree of clinical suspicion and is supported by CSF studies showing a modest pleocytosis, elevated protein, and reactive VDRL.

